# Recycled Fine Aggregates from Mortar Debris and Red Clay Brick to Fabricate Masonry Mortars: Mechanical Analysis

**DOI:** 10.3390/ma15217707

**Published:** 2022-11-02

**Authors:** René Sebastián Mora-Ortiz, Sergio Alberto Díaz, Ebelia Del Angel-Meraz, Francisco Magaña-Hernández

**Affiliations:** División Académica de Ingeniería y Arquitectura (DAIA), Universidad Juárez Autónoma de Tabasco, Carretera Cunduacán-Jalpa de Méndez km. 1, Cunduacán 86690, Tabasco, Mexico

**Keywords:** construction and demolition waste, recycled aggregates, sustainable construction, mortars, red clay brick waste

## Abstract

In this research, the mechanical behavior of masonry mortars made with partial substitution of sand by recycled fine aggregates (RFAs) of mortar (MT) and recycled clay brick (RCB) was compared. Mortar specimens were built in two groups (MT and RCB) considering different replacement proportions by dry weight. To reduce the water absorption of RFAs during mortar making, the prewetting method was utilized. All the mixtures were assembled with a volumetric cement-to-aggregate ratio of 1:4 and a consistency of 175 ± 5 mm. The properties in the fresh and hardening state of mortars were analyzed separately. The experimental results showed that the properties of mortars in a fresh state (bulk density and air content) were affected if RFA was added to the mixture; however, mortars assembled with up to 40% and 50% of MT and RCB, respectively, accomplished a compressive strength value of reference for new mixtures. Both mortar groups showed good results in adhesive strength values, with the RCB mortars standing up as they achieved greater adherence than the control mortar with substitution percentages of up to 30%. Therefore, the reutilization of both RFAs is feasible, notably in rendering and bonding functions.

## 1. Introduction

In recent years, the sustainable engineering of construction development has increased; therefore, several researchers have doubled their efforts toward projects where the mean objective is to analyze the feasibility of recycling construction and demolition waste (CDW) as new building materials, focusing on mechanical behavior, durability, and environmental impact [1,2,3,4,5,6]. One way these residues can be used is as partial substitutes for natural aggregates (NAs) in concrete and mortar mixes, such as recycled coarse aggregates (RCAs) and recycled fine aggregates (RFAs) [7].

Due to many countries having restrictive construction laws regarding recycled aggregates (RAs), the use of RA is mainly focused on nonstructural purposes; therefore, its recycling as a partial natural aggregate replacement in masonry mortar mixtures represents a feasible option [8,9,10]. In this way, one obstacle to RFA recycling has been the characteristics of the material itself: high porosity and high-water absorption [11,12,13]. Consequently, in recent years, investigations have been developed about how these materials can be used as new aggregates if they are processed following appropriate deconstruction procedures and if they are properly pretreated. Deconstruction strategies or selective demolition strategies allow obtaining a good-quality RFA with a reduced quantity of contaminants [4,14,15,16,17,18]. Coelho et al. [19] and Kumbhar et al. [20] described some selective demolition strategies. With regard to RFA pretreatment, prewetting the aggregate is a process that improves behavior; it is easy to implement, it is cheap, and it is environmentally friendly [21,22,23]. Various researchers have proven that the prewetting process of RAs reduces the amount of water needed to create a mortar mixture, improving the workability and compression resistance of concrete [22,24,25] and mortar [23,26,27] samples. Cuenca-Moyano et al. [28], Cabral et al. [29], Zhao et al. [30], and Etxeberria et al. [31] recommended prewetting the RA to 80% of its entire absorption capacity for best performance.

The RFAs from concrete debris have been deeply analyzed, and several researchers have proven their usefulness in masonry mortar elaboration with up to 25% content [32,33,34,35]. In the last few decades, the recycling of other types of materials as a partial substitute for NA in mortar mixtures has stolen the attention of researchers. Silva et al. [36] concluded that preparing mortar with 10% crushed ceramic improved the mortar properties. Oliveira et al. [37] built mortars with a partial substitution for NA using different percentages (25%, 50%, 75%, and 100%) of ceramic recycled aggregate (CRA). They observed that mortars with CRA showed less compressive strength and higher adhesive strength values in comparison with conventional mortar. Martínez et al. [38] and Rubio de Hita et al. [39] reached similar conclusions.

A material that is part of CDW and is not adequately recycled is that originating from red clay brick. Debieb and Kenai [40] utilized fine and coarse aggregates of crushed bricks to create new mortar mixes, and they concluded the suitability of 25% and 50% as the maximum percentages of exploitation for thick aggregate and fine aggregate, respectively. Bektas et al. [41] used recycled clay brick as fine aggregate in mortar mixtures, with substitution percentages of 10% and 20% dry weight. These researchers reported no negative effects due to the substitution percentages used, along with a minor influence on the mortar shrinkage. Silva et al. [42] studied the most favorable substitution percentages of RFA from red clay bricks instead of NA, with 20% identified as the most acceptable value. Corinaldesi [43] utilized fine crushed red brick in the production of mortars and concluded that, even when the fine aggregate shows inferior mechanical properties, it enhances mortar adherence. Zhi et al. [44] concluded that resistance to drying shrinkage improved with the increase in prewetted fine clay brick, as a result of the release of prestored water in the aggregate.

A poorly studied material is the RFA from mortar debris. This is because, when compared with other RFAs, it shows lower quality. However, researchers such as Jiménez et al. [45] observed that using RFA composed of ceramic and mortar as a fine aggregate in new mortar mixes, with a 40% maximum substitution percentage, did not considerably affect the mortar properties, except for its density and workability. Mora-Ortiz et al. [46] demonstrated that, if the aggregate is obtained through a well-planned demolition strategy while using commercial plasticizers, it can be successfully reutilized in mortar mixes with a maximum percentage substitution of 40%.

The aim of this paper was to separately analyze the mechanical behavior of mortar mixes created with RFAs from mortar debris and red clay bricks, as a partial substitution of NA. The expectation was that, using a selective demolition and prewetting process, both RFA types could be used as NA partial substitutes in mortar mixes.

## 2. Materials and Methods

### 2.1. Recycled Fine Aggregates

**Figure 1:**
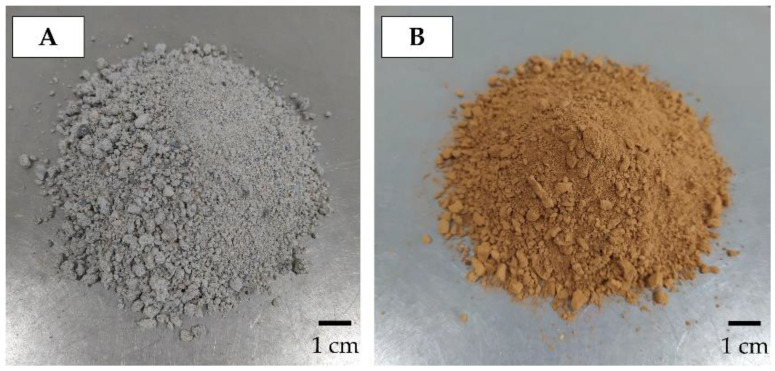
Recycled fines aggregates of (**A**) mortar and (**B**) red clay brick.

The RFAs used in this investigation were obtained from a demolition site localized in the city of Cunduacán, Tabasco, México. The demolished structure was a single-family home with one level of construction. It had some interior walls built with red clay bricks and others with mortar blocks. As part of the strategy to take advantage of the debris, while reducing as much as possible the risk of contaminating the aggregates on the site, a deconstruction procedure was followed. This procedure was detailed in Mora-Ortiz et al. [47] and consisted of removing the maximum amount of painting or any wall covering that could contaminate the debris (wood, metal, plastics, etc.); subsequently, the structural elements of the house were demolished, and the debris was carefully stored in a clean and adequate place for this purpose. This deconstruction procedure was executed on structural elements with red clay brick and mortar blocks, classified by origin, into two different storage containers. After debris collection, all the recycled material was crushed and separated using a Los Angeles abrasion machine before screening. According to the above procedure, recycled fines aggregates (RFAs) were obtained from red clay brick (RCB) and mortar (MT) (Figure 1).

### 2.2. Characterization of the Materials

**Figure 2:**
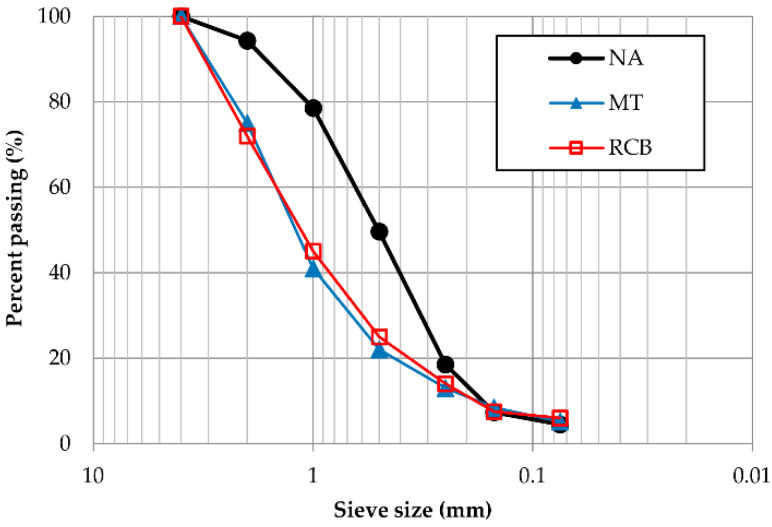
Particle size distribution of aggregates (UNE-EN 933-1:2012 [48]).

Figure 2 and Table 1 show the granulometric curves and characteristics of the studied aggregates in this research. The natural aggregate (NA) was river sand from Samaria’s bank, in the city of Cunduacán, Tabasco, México. As shown in Table 1, the sulfate and chloride composition of the aggregates were as expected, despite the RCB showing relatively higher content due to the primary element of this sample, i.e., clay.

**Figure 3:**
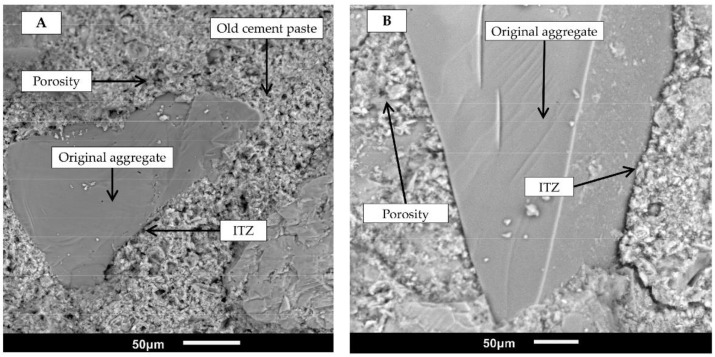
SEM photomicrograph of (**A**) recycled mortar aggregate and (**B**) recycled red clay brick aggregate.

Both recycled aggregates had better water absorption than NA. This is because both materials had greater porosity and more interfacial transition zones (ITZs) between the fine aggregate and original cement paste [52]. These characteristics also resulted in a lower particle density of RAs than NA (Table 1). Figure 3 shows two scanning electron microscope (SEM) images at a scale of 50 μm, taken using a JEOL JSM6010LA electronic microscope (Boston, MA, USA), which correspond to recycled mortar aggregate and recycled red clay brick aggregate. Both cases show the high level of porosity and the increased presence of ITZs.

**Figure 4:**
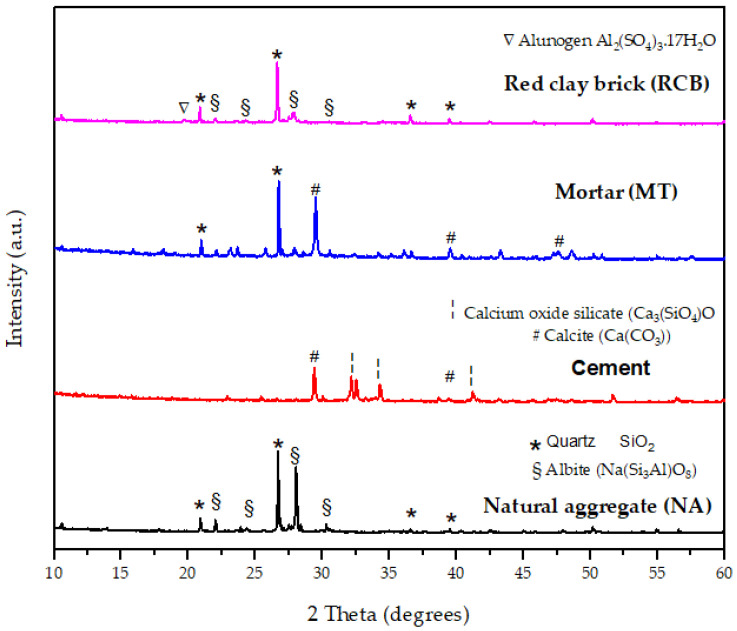
X-ray diffraction patterns for the cement and aggregates used.

To identify the mineralogical characteristics of aggregates, an X-ray diffraction analysis was executed over pulverized aggregate samples, using Cu-Kα (𝜆 = 0.154 nm) radiation, a 10°–60° scanning range with a 0.02° step size, and 0.3 s of transition time. Figure 4 shows the X-ray diffraction patterns for the cement and aggregates.

The different structural phases found were quartz (PDF 00-046-1045), calcite (PDF 04-008-0198), albite (PDF 00-010-0393), calcium oxide silicate (PDF 04-018-9701), calcium aluminum silicate (PDF 00-052-1344), calcium magnesium iron carbonate (PDF 04-023-8806), rubidium bismuth molybdenum oxide (PDF 05-001-0380), aluminum sulfate hydrate known as alunogen (PDF 00-022-0022), and amorphous silica. Red clay brick was a combination of quartz, the feldspar albite, and a small trace of alunogen; quartz was the dominant material due to the thermal treatment applied in its cooking process. Likewise, the recycled mortar aggregate mainly constituted quartz and calcite. The cement utilized was CEMEX PPC 30R type, commercialized in México [53,54], with a specific gravity of 3.15 kg/m^3^. Its chemical composition is shown in Table 2.

### 2.3. RFA Prewetting Method

As mentioned above, one of the drawbacks of using CDW in mortar mixture production is its high water absorption. To reduce this absorption, the prewetting technique described by Cuenca Moyano et al. [23] was applied. Briefly, in a standard mixer, the RAs and distilled water were mixed at low speed for 5 min; subsequently, the RAs were left to rest submerged for 10 min, after which they were removed from the water and allowed to drain before use. During this procedure, the RFAs were prewetted with up to 67% of their absorption capacity (WA_24h_), which considerably reduced the amount of water taken from the cement paste for the recycled fine aggregate.

### 2.4. Mixes

The dosage for a regular masonry mortar (cement + sand + water) was designed using a 1:4 cement–sand ratio as a control mixture. With reference to the control mixture dosage, two mortar mixtures were created using recycled mortar aggregate (MT) or red clay recycled aggregate (RCB) as a partial substitute for NA. The mixtures were classified according to their substitution percentages: 10%, 20%, 30%, 40%, 50%, 60%, 80%, and 100% according to dry weight [23,28,45,55]. The project consistency used in all mixtures was 175 ± 5 mm. Table 3 shows the nomenclature and proportions used for the mortars.

### 2.5. Rehearsal Program

To evaluate the mechanical behavior of the mortars in this investigation, the mortar mixtures were analyzed in fresh and hardened states. Table 4 shows the standards of reference for the analyzed properties. The curing conditions were as follows: in the first 2 days, the mortar samples were introduced in a chamber at a temperature of 20 ± 2 °C and a relative humidity of 95% ± 5%. Subsequently, they were demolded and maintained under the same temperature and humidity conditions. After 7 days, the relative humidity was reduced to 65% ± 5% until testing.

## 3. Results and Discussion

### 3.1. Fresh Mortar

#### 3.1.1. Bulk Density

**Figure 5:**
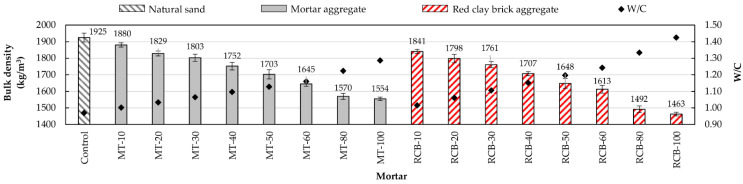
Bulk density variation of mortars.

**Figure 6:**
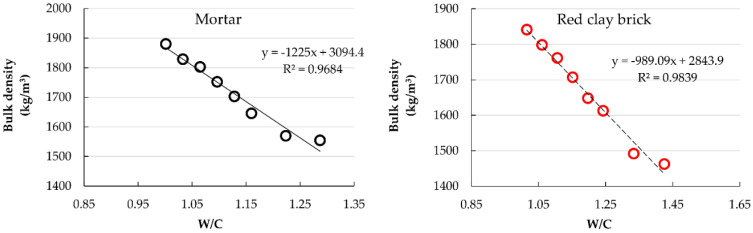
Evolution on bulk density with changes in W/C ratio.

As can be seen in Figure 5, with the increase in RFA substitution percentage for natural sand, the bulk density decreased, whereby mortars with a lower density had a higher substitution percentage. This behavior was repeated in both recycled mortar groups (MT and RCB), with RCB mortars suffering the largest decrease in density. For example, with a 10% substitution ratio, the mortar mixture created using recycled mortar aggregates (MT-10) showed a density decrease of around 2.34% with respect to the control mortar, whereas mortars made using red clay brick recycled aggregate (RCB-10) showed a bigger loss of density (4.42%). This decrease in density was due to two factors: the high water absorption of RFAs and the low density of particles [13,42,62]. Figure 6 shows the good correlation between bulk density and water/cement ratio (W/C).

#### 3.1.2. Air Content

There is currently no reference standard for air content limit values; hence, in this investigation, the criteria proposed by Cuenca-Moyano et al. [23,28] were adapted. These criteria suggest that the optimum air content is between 5% and 20%.

**Figure 7:**
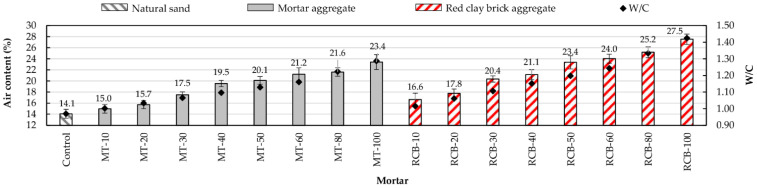
Air content variation with changes in W/C ratio.

Figure 7 shows that, in both mortar groups, the air content increased with the RFA content. Comparing RCB mortars with their counterpart MT mortars, the former remarkably reached higher air content values at all substitution proportions. due to the greater water absorption of the recycled red clay brick (RCB) aggregate (Table 1).

Mortars of the MT group manufactured with substitution percentages ≤40% satisfied the criteria established in this investigation. On the other hand, only RCB mortars with substitution percentages ≤20% accomplish the criteria.

**Figure 8:**
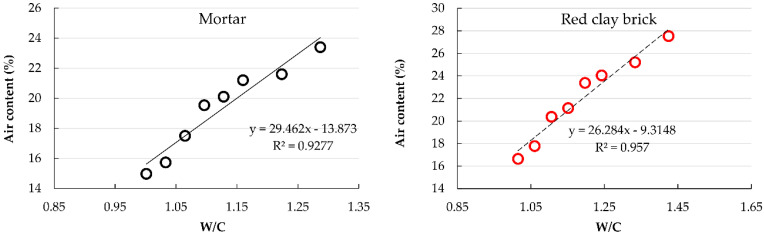
Evolution of air content with changes in W/C ratio.

Figure 8 shows the relationship between air content and water/cement ratio.

Following the characterization of the fresh state mortars, a close affinity was observed between the mortar properties and the water/cement ratio; an increase in W/C ratio led to an increase in air content but a decrease in density of the fresh state mortars. This behavior followed the trend found by other researchers using several types of RFA in concrete [3,63] and mortar [37,45,64].

### 3.2. Hardened Mortar

#### 3.2.1. Dry Bulk Density

**Figure 9:**
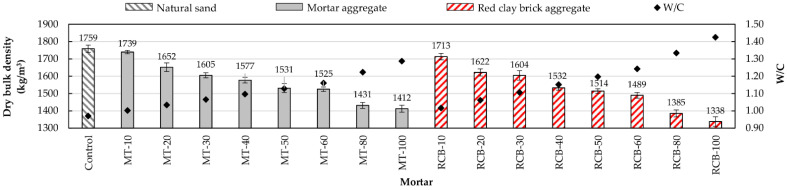
Dry bulk density variation with changes in W/C ratio.

The changes in dry bulk density with the increment in W/C ratio for both groups of mortars are shown in Figure 9. It can be observed that the dry bulk density in all mortars decreased with the increment in RFA. As previously mentioned, this was due to the porosity and high water absorption of the RFAs. These observations matched those of researchers using RFAs obtained from ceramics [45,65] and concrete [30,33,66].

Comparing the densities achieved by the mortars of both groups, it can be observed that those made using red clay brick (RCB) developed lower densities due to their lower specific density and higher water absorption.

**Figure 10:**
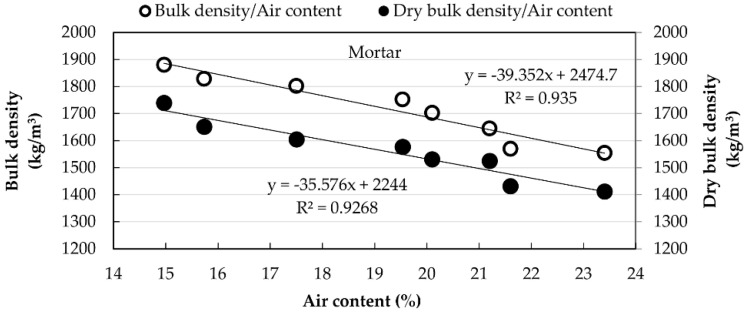
Relationship between densities of MT mortars and air content.

**Figure 11:**
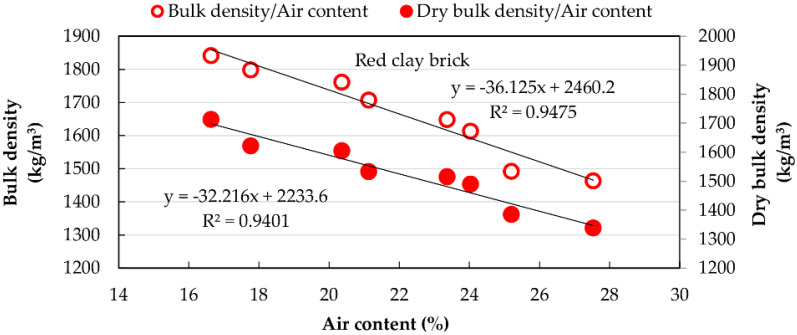
Relationship between densities of RCB mortars and air content.

Figure 10 and Figure 11 show the relationship between air content and densities, revealing a decrease in densities with the increase in air content for both materials.

#### 3.2.2. Adhesive Strength

**Figure 12:**
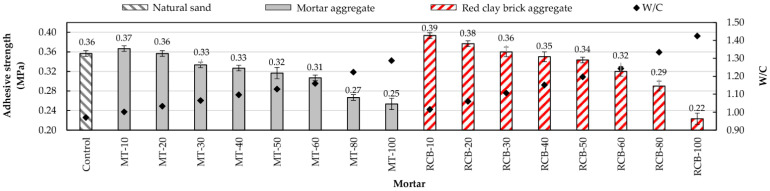
Adhesive strength and W/C ratios of mortars.

The adhesive strength of each mortar is shown in Figure 12. In general, all mortars presented a similar behavior, whereby adherence decreased with the increases in RFA content and W/C ratio. However, it was observed that, except for mortars with 100% substitution, the adherence of RCB mortars was the greatest. This is interesting as RCB mortars have so far exhibited the most unfavorable values in the properties analyzed.

Contrasting the mortars made using RFA with the control mortar (reference value), those of the MT group with 10% and 20% substitution values exhibited equal or superior adherence, whereas the mortars of the RCB group with 10%, 20%, and 30% substitution values exhibited equal or superior values. This behavior was previously observed by researchers using RFAs from concrete [33,67] and ceramics [37,45]. Additionally, it was observed that the RCB mortar with 40% substitution reached values very close to the control mortar, indicating that this substitution percentage did not considerably influence the adhesive strength of the mortar.

To explain this behavior, it is necessary to remember that RFAs have high porosity and increased interfacial transition zones (ITZs) by nature (Figure 3), resulting in high water absorption and low density (Table 1). During the grinding process, the surface of the RFAs is irregular and rough, featuring many edges [37,68]. These characteristics are detrimental to some mortar properties but useful in improving adhesive strength, whereby cement pastes exhibit greater adherence with the aggregate while infiltrating the pores and the ITZ, acting as an anchor [69,70]. Silva et al. [42], Oliveira et al. [37], Martínez et al. [71], Corinaldesi [43], and Jiménez et al. [72] reported this behavior in mortar made using ceramic material.

#### 3.2.3. Compressive Strength

**Figure 13:**
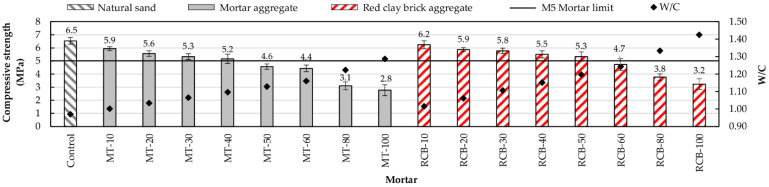
Compressive strength and W/C ratios of mortars.

The compressive strengths developed by all mortars are shown in Figure 13. A similar trend to that in previous sections was observed, revealing a correspondence between the strength and RFA content. As the substitution percentage increased, the W/C ratio increased while the strength decreased. These results match those observed in other investigations [21,28,37,65].

To analyze the resistance of the mortars, the compressive strength of class M5 mortar was used as a reference. The mortars of the MT group with up to 50% substitution no longer reached the reference resistance, whereas the mortars of the RCB group with up to 60% substitution no longer reached the reference resistance. Comparing the corresponding mortars of both groups, it can be seen that the RCB mortars developed a higher compressive strength. For example, the RCB-50 mortar exhibited 15.22% more toughness than its MT-50 counterpart. On the other hand, the RCB-10 mortar exhibited a resistance very similar to the reference mortar (control), indicating that substitution percentage did not influence this property.

It should be noted that the highest resistance across both groups of mortars was achieved by the RCB mortars due to the penetration of the cement paste into the pores and the ITZ, as well as its better adhesion to the aggregate surface. Researchers such as Vegas et al. [32] and Farinha et al. [73] have previously pointed out the possibility of the small RFA particles exerting a slight pozzolanic effect [74,75].

#### 3.2.4. Water Absorption Due to Capillary Action of Hardened Mortar

**Figure 14:**
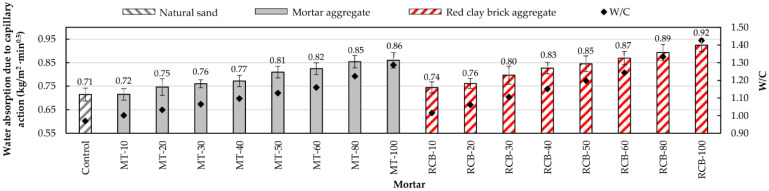
Water absorption due to capillary action of mortars.

**Figure 15:**
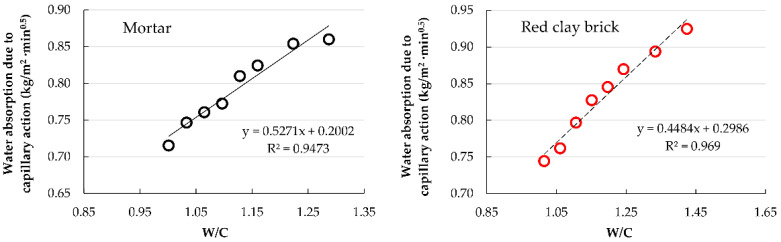
Relationship between water absorption due to capillary action and changes in the W/C ratio.

In Figure 14, it can be observed that the water absorption via capillary action increased with the substitution percentage of RFA in both groups of mortars. Accordingly, mortars with the highest absorption were those with 100% substitution. Comparing the mortars of both groups, it can be noted that the RCB mortars showed higher values of water absorption via capillary action than their MT counterparts due to their higher W/C ratio [28,45]. The relationship between water absorption via capillary action and changes in W/C ratio are shown in Figure 15.

This property is one of the main indicators of the durability of mortars [23,67]. This is because high absorption values correspond to more developed pore networks and, consequently, a greater possibility of contaminants flowing into the interior of the mortar; therefore, high absorption values correspond to low durability.

#### 3.2.5. Susceptibility to Cracking

This test was performed according to the procedure described by Oliveira et al. [76], Farinha et al. [73], and Sara et al. [77]. It consisted of the application of a 2 cm thick mortar layer to a ceramic brick. Three specimens of each mortar were utilized and visually evaluated for 120 days to evaluate the occurrence of cracking.

**Figure 16:**
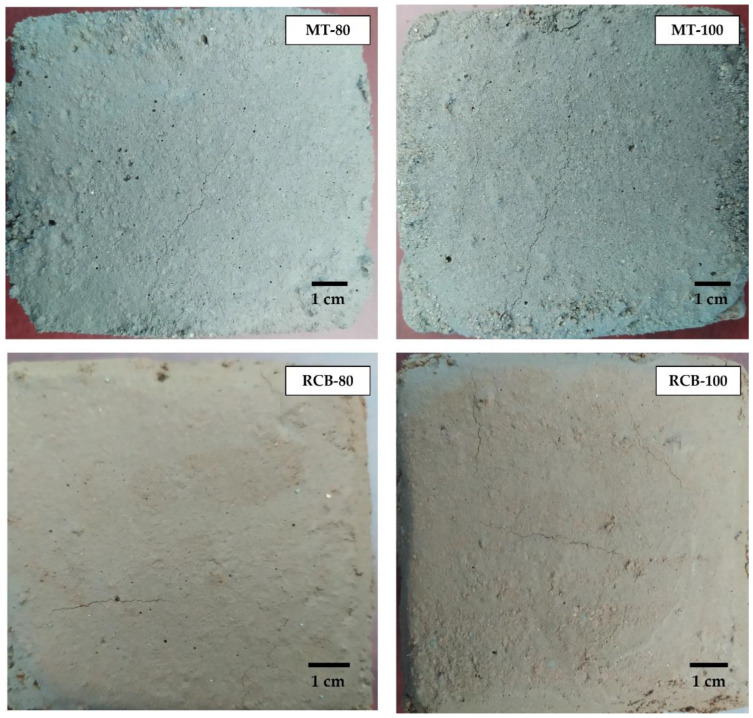
Visible cracks in mortars with 80% and 100% substitution. MT = mortar; RCB = red clay brick.

As a result, only mortars with 80% and 100% substitution showed visible cracks in both cases (Figure 16). The prewetting process utilized in this paper contributed to a reduction in water absorption of the RFA, hydrating it and reducing the transfer of water between the RFA and cement paste [23,28], thus helping to prevent cracks.

Farinha et al. [73], Oliveira et al. [76], and Braga et al. [78] analyzed the behavior of mortars made using 10%, 15%, and 20% substitution percentages of RFAs, and found that none of the mortars showed visible cracks. Thus, prewetting contributes to a reduction in cracking at low and medium replacement levels.

## 4. Conclusions

The gradual incorporation of MT and RCB RFAs into mortars resulted in an increase in W/C ratio, thus influencing properties such as density. The increase in water content is a natural effect of the RFAs due to their greater porosity than NA, in addition to the development of an ITZ between the cement paste and the original aggregate. As the RCB aggregate had a higher water absorption than the MT aggregate, it exhibited the most unfavorable values with regard to the aforementioned properties.

Nevertheless, except for those with 100% replacement of sand by RFA, all RCB mortars showed the highest adhesion values across both groups of mortars. Even those with up to 30% substitution exceeded the adhesion value of the control mortar, due to the greater porosity and the increased presence of ITZs. These characteristics simultaneously impaired the air content, bulk density, and dry bulk density of the mortars.

Regarding the compressive strength, both groups of mortars showed good performance up to 40% replacement, exceeding the reference resistance. The water absorption values due to capillary action in both groups of mortars were higher than those shown by the control mortar, particularly for RCB mortars. However, in the susceptibility to cracking test, no visible cracks were observed for mortars of both groups with up to 80% substitution. This was due to the prewetting process used in the RFA.

Taken altogether, we can conclude that, using the processes described in this research, it may be feasible to recycle RFAs from MT and RCB as partial substitutes for sand (20% and 30%, respectively) in masonry mortars for indoor functions, i.e., rendering and bonding. For outdoor functions, it is necessary to carry out more detailed durability studies.

## Figures and Tables

**Table 1:** Main characteristics of aggregates.

Property	Standard	Limit Value	NA	MT	RCB
Fine content (%)	EN 933-1 [48]	≤30	4.5	5.2	6.0
Sand equivalent (%)	EN 933-8 [49]	No limit	94	82	84
Dry sample density (g/cm^3^)	EN 1097-6 [50]	No limit	2.66	2.47	2.31
Water absorption (%)	EN 1097-6 [50]	No limit	1.25	7.46	10.74
Acid soluble sulfates (% SO_3_)	EN 1744-1 [51]	≤0.8	<0.010	0.0053	0.0061
Water-soluble chlorides (% Cl)	EN 1744-1 [51]	≤0.06	<0.010	0.030	0.038
Total sulfurs (% SO_3_)	EN 1744-1 [51]	≤1	<0.010	0.0057	0.0068

**Table 2:** Chemical composition of cement used.

Composition	CaO	SiO_2_	Al_2_O_3_	Fe_2_O_3_	MgO	K_2_O	Na_2_O	SO_3_
%	63	22	6	2.5	2.6	0.6	0.3	2.0

**Table 3:** Mortar mixture proportions.

Mortar Type	Aggregate Type	NA/RA (%)	NA (g)	RA (g)	CEM (g)	Prewetting Water (g)	Mixing Water (g)	Total Water (g)	Consistency Index (mm)	W/C
Control	Natural aggregate	100/0	2200	0	495	0	480	480	174	0.970
MT-10	Mortar	90/10	1980	220	495	11	480	491	179	0.992
MT-20		80/20	1760	440	495	22	480	502	171	1.014
MT-30		70/30	1540	660	495	33	480	513	178	1.036
MT-40		60/40	1320	880	495	44	480	524	172	1.059
MT-50		50/50	1100	1100	495	55	480	535	180	1.081
MT-60		40/60	880	1320	495	66	480	546	173	1.103
MT-80		20/80	440	1760	495	88	480	568	170	1.147
MT-100		0/100	0	2200	495	110	480	590	177	1.192
RCB-10	Red clay brick	90/10	1980	220	495	16	480	496	179	1.002
RCB-20		80/20	1760	440	495	32	480	512	175	1.034
RCB-30		70/30	1540	660	495	47	480	527	172	1.066
RCB-40		60/40	1320	880	495	63	480	543	174	1.098
RCB-50		50/50	1100	1100	495	79	480	559	173	1.130
RCB-60		40/60	880	1320	495	95	480	575	176	1.162
RCB-80		20/80	440	1760	495	127	480	607	176	1.226
RCB-100		0/100	0	2200	495	158	480	638	171	1.290

W/C: water/cement radio; CEM: cement.

**Table 4:** Standards of reference utilized.

Test	Standard	Specimens and Dimensions	Curing Time (Days)
*Fresh mortar*			
Bulk density of the fresh mortar	UNE-EN 1015-6 [56]	3	---
Entrained air	UNE-EN 1015-7 [57]	3	---
*Hardened mortar*			
Dry bulk density	UNE-EN 1015-10 [58]	3 (40 × 40 × 160 mm)	28
Compressive strength	UNE-EN 1015-11 [59]	3 (40 × 40 × 80 mm)	28
Adhesive strength	UNE-EN 1015-12 [60]	3 (50 mm diameter, 10 mm thick)	28
Water absorption coefficient due to capillary action	UNE-EN 1015-18 [61]	3 (40 × 40 × 80 mm)	28
Susceptibility to cracking	---	2 (layer of mortar 2 cm thick in a brick)	120

## Data Availability

Data are contained within the article.

## References

[1] Zhang J., Ding L., Li F., Peng J. (2020). Recycled aggregates from construction and demolition wastes as alternative filling materials for highway subgrades in China. J. Clean. Prod..

[2] Mah C.M., Fujiwara T., Ho C.S. (2018). Life cycle assessment and life cycle costing toward eco-efficiency concrete waste management in Malaysia. J. Clean. Prod..

[3] Kim J., Grabiec A.M., Ubysz A. (2022). An Experimental Study on Structural Concrete Containing Recycled Aggregates and Powder from Construction and Demolition Waste. Materials.

[4] Colangelo F., Petrillo A., Farina I. (2021). Comparative environmental evaluation of recycled aggregates from construction and demolition wastes in Italy. Sci. Total Environ..

[5] Contreras-Llanes M., Romero M., Gázquez M.J., Bolívar J.P. (2021). Recycled Aggregates from Construction and Demolition Waste in the Manufacture of Urban Pavements. Materials.

[6] Kirthika S.K., Singh S.K., Chourasia A. (2020). Performance of Recycled Fine-Aggregate Concrete Using Novel Mix-Proportioning Method. J. Mater. Civ. Eng..

[7] Bu C., Liu L., Lu X., Zhu D., Sun Y., Yu L., OuYang Y., Cao X., Wei Q. (2022). The Durability of Recycled Fine Aggregate Concrete: A Review. Materials.

[8] Ferreira R.L., Anjos M.A., Nóbrega A.K., Pereira J.E.S., Ledesma E.F. (2019). The role of powder content of the recycled aggregates of CDW in the behaviour of rendering mortars. Constr. Build. Mater..

[9] Martínez I., Etxeberria M., Pavón E., Díaz N. (2018). Influence of Demolition Waste Fine Particles on the Properties of Recycled Aggregate Masonry Mortar. Int. J. Civ. Eng..

[10] Fan C.-C., Huang R., Hwang H., Chao S.-J. (2015). The Effects of Different Fine Recycled Concrete Aggregates on the Properties of Mortar. Materials.

[11] Feng P., Chang H., Xu G., Liu Q., Jin Z., Liu J. (2019). Feasibility of Utilizing Recycled Aggregate Concrete for Revetment Construction of the Lower Yellow River. Materials.

[12] Nili M., Sasanipour H., Aslani F. (2019). The Effect of Fine and Coarse Recycled Aggregates on Fresh and Mechanical Properties of Self-Compacting Concrete. Materials.

[13] Li Z., Liu J., Xiao J., Zhong P. (2018). A method to determine water absorption of recycled fine aggregate in paste for design and quality control of fresh mortar. Constr. Build. Mater..

[14] Rahal K. (2007). Mechanical properties of concrete with recycled coarse aggregate. Build. Environ..

[15] Cachim P.B. (2009). Mechanical properties of brick aggregate concrete. Constr. Build. Mater..

[16] Debieb F., Courard L., Kenai S., Degeimbre R. (2009). Roller compacted concrete with contaminated recycled aggregates. Constr. Build. Mater..

[17] Akbarnezhad A., Ong K.C.G., Chandra L.R. (2014). Economic and environmental assessment of deconstruction strategies using building information modeling. Autom. Constr..

[18] Rao M.C., Bhattacharyya S.K., Barai S.V. (2019). Demolition Techniques and Production of Recycled Aggregate.

[19] Coelho A., De brito J. (2013). Conventional Demolition versus Deconstruction Techniques in Managing Construction and Demolition Waste (CDW). Handbook of Recycled Concrete and Demolition Waste.

[20] Kumbhar S.A., Gupta A., Desai D.B. (2013). Recycling and Reuse of Construction and Demolition Waste for Sustainable Development. Int. J. Sustain. Dev..

[21] Mefteh H., Kebaili O., Oucief H., Berredjem L., Arabi N. (2013). Influence of moisture conditioning of recycled aggregates on the properties of fresh and hardened concrete. J. Clean. Prod..

[22] Sánchez-Roldán Z., Martín-Morales M., Valverde-Palacios I., Valverde-Espinosa I., Zamorano M. (2016). Study of potential advantages of pre-soaking on the properties of pre-cast concrete made with recycled coarse aggregate. Mater. Constr..

[23] Cuenca-Moyano G.M., Martín-Morales M., Valverde-Palacios I., Valverde-Espinosa I., Zamorano M. (2014). Influence of pre-soaked recycled fine aggregate on the properties of masonry mortar. Constr. Build. Mater..

[24] González J.G., Robles D.R., Valdés A.J., del Pozo J.M.M., Romero M.I.G. (2013). Influence of Moisture States of Recycled Coarse Aggregates on the Slump Test. Adv. Mater. Res..

[25] Rashid K., Rehman M.U., de Brito J., Ghafoor H. (2020). Multi-criteria optimization of recycled aggregate concrete mixes. J. Clean. Prod..

[26] Jochem L.F., Aponte D., Bizinotto M.B., Rocha J.C. (2019). Effects of pre-wetting aggregate on the properties of mortars made with recycled concrete and lightweight aggregates. Matéria (Rio Janeiro).

[27] Kim J.-H., Robertson R.E. (1997). Prevention of air void formation in polymer-modified cement mortar by pre-wetting. Cem. Concr. Res..

[28] Cuenca-Moyano G.M., Martín-Pascual J., Martín-Morales M., Valverde-Palacios I., Zamorano M. (2020). Effects of water to cement ratio, recycled fine aggregate and air entraining/plasticizer admixture on masonry mortar properties. Constr. Build. Mater..

[29] Cabral A.E.B., Schalch V., Molin D.C.C.D., Ribeiro J.L.D. (2010). Mechanical properties modeling of recycled aggregate concrete. Constr. Build. Mater..

[30] Zhao Z., Remond S., Damidot D., Xu W. (2015). Influence of fine recycled concrete aggregates on the properties of mortars. Constr. Build. Mater..

[31] Etxeberria M., Vázquez E., Mari A., Barra M. (2007). Influence of amount of recycled coarse aggregates and production process on properties of recycled aggregate concrete. Cem. Concr. Res..

[32] Vegas I., Azkarate I., Juarrero A., Frías M. (2009). Diseño y prestaciones de morteros de albañilería elaborados con áridos reciclados procedentes de escombro de hormigón. Mater. Construcción.

[33] Neno C., De Brito J., Veiga R. (2014). Using Fine Recycled Concrete Aggregate for Mortar Production. Mater. Res..

[34] Saiz-Martínez P., González-Cortina M., Fernández-Martínez F. (2015). Characterization and Influence of Fine Recycled Aggregates on Masonry Mortars Properties. Mater. Construcción.

[35] Ng S., Engelsen C.J. (2018). Construction and demolition wastes. Waste and Supplementary Cementitious Materials in Concrete.

[36] Silva J., de Brito J., Veiga R. (2009). Incorporation of fine ceramics in mortars. Constr. Build. Mater..

[37] de Oliveira Andrade J.J., Possan E., Squiavon J.Z., Ortolan T.L.P. (2018). Evaluation of mechanical properties and carbonation of mortars produced with construction and demolition waste. Constr. Build. Mater..

[38] Martínez I., Etxeberria M., Pavón E., Díaz N. (2013). A comparative analysis of the properties of recycled and natural aggregate in masonry mortars. Constr. Build. Mater..

[39] De Hita P.R., Pérez-Gálvez F., Morales-Conde M.J., Pedreño-Rojas M.A. (2019). Characterisation of recycled ceramic mortars for use in prefabricated beam-filling pieces in structural floors. Mater. Construcción.

[40] Debieb F., Kenai S. (2008). The use of coarse and fine crushed bricks as aggregate in concrete. Constr. Build. Mater..

[41] Bektas F., Wang K., Ceylan H. (2009). Effects of crushed clay brick aggregate on mortar durability. Constr. Build. Mater..

[42] Silva J., de Brito J., Veiga R. (2010). Recycled Red-Clay Ceramic Construction and Demolition Waste for Mortars Production. J. Mater. Civ. Eng..

[43] Corinaldesi V. (2012). Environmentally-friendly bedding mortars for repair of historical buildings. Constr. Build. Mater..

[44] Ge Z., Feng Y., Zhang H., Xiao J., Sun R., Liu X. (2020). Use of recycled fine clay brick aggregate as internal curing agent for low water to cement ratio mortar. Constr. Build. Mater..

[45] Jiménez J.R., Ayuso J., López M., Fernández J.M., De Brito J.M.C.L. (2013). Use of fine recycled aggregates from ceramic waste in masonry mortar manufacturing. Constr. Build. Mater..

[46] Mora-Ortiz R.S., Munguía-Balvanera E., Díaz S.A., Magaña-Hernández F., Del Angel-Meraz E., Bolaina-Juárez Á. (2020). Mechanical Behavior of Masonry Mortars Made with Recycled Mortar Aggregate. Materials.

[47] Mora-Ortiz R.S., Del Angel-Meraz E., Díaz S., Magaña-Hernández F., Munguía-Balvanera E., Castro M.P., Alavez-Ramírez J., Quiroga L.A. (2021). Effect of Pre-Wetting Recycled Mortar Aggregate on the Mechanical Properties of Masonry Mortar. Materials.

[48] (2012). Ensayos Para Determinar Las Propiedades Geométricas de Los Áridos. Parte 1: Determinación De La Granulometría De Las Partículas. Método Del Tamizado.

[49] (2015). Ensayos Para Determinar Las Propiedades Geométricas De Los Áridos. Parte 8: Evaluación De Los Finos. Ensayo Del Equivalente De Arena.

[50] (2014). Ensayos Para Determinar Las Propiedades Mecánicas Y Físicas De Los Áridos. Parte 6: Determinación De La Densidad De Partículas Y La Absorción De Agua.

[51] (2013). Ensayos Para Determinar Las Propiedades Químicas De Los Áridos. Parte 1: Análisis Químico.

[52] Yang J., Du Q., Bao Y. (2011). Concrete with recycled concrete aggregate and crushed clay bricks. Constr. Build. Mater..

[53] (2019). Standard Specification for Portland Cement.

[54] (2021). Standard Specification for Blended Hydraulic Cements.

[55] Evangelista L., de Brito J. (2007). Mechanical behaviour of concrete made with fine recycled concrete aggregates. Cem. Concr. Compos..

[56] (2007). Métodos De Ensayo De Los Morteros Para Albañilería. Parte 6: Determinación De La Densidad Aparente Del Mortero Fresco.

[57] (1999). Métodos De Ensayo De Los Morteros Para Albañilería. Parte 7: Determinación Del Contenido En Aire En El Mortero Fresco.

[58] (2000). Métodos De Ensayo De Los Morteros Para Albañilería. Parte 10: Determinación De La Densidad Aparente En Seco Del Mortero Endurecido.

[59] (2000). Métodos De Ensayo De Los Morteros Para Albañilería. Parte 11: Determinación De La Resistencia A Flexión Y A Compresión Del Mortero Endurecido.

[60] (2016). Métodos De Ensayo De Los Morteros Para Albañilería. Parte 12: Determinación De La Resistencia A La Adhesión De Los Morteros De Revoco Y Enlucido Endurecidos Aplicados Sobre Soportes.

[61] (2003). Métodos De Ensayo De Los Morteros Para Albañilería. Parte 18: Determinación Del Coeficiente De Absorción De Agua Por Capilaridad Del Mortero Endurecido.

[62] Cartuxo F., de Brito J., Evangelista L., Jiménez J.R., Ledesma E.F. (2016). Increased Durability of Concrete Made with Fine Recycled Concrete Aggregates Using Superplasticizers. Materials.

[63] Leite M.B., Figueire do Filho J.G.L., Lima P.R.L. (2013). Workability study of concretes made with recycled mortar aggregate. Mater. Struct..

[64] Małek M., Łasica W., Jackowski M., Kadela M. (2020). Effect of Waste Glass Addition as a Replacement for Fine Aggregate on Properties of Mortar. Materials.

[65] Ledesma E.F., Jiménez J.R., Ayuso J., Fernández J.M., de Brito J. (2015). Maximum feasible use of recycled sand from construction and demolition waste for eco-mortar production—Part-I: Ceramic masonry waste. J. Clean. Prod..

[66] Ledesma E.F., Jiménez J.R., Fernández J., Galvín A.P., Agrela F., Barbudo M.A. (2014). Properties of masonry mortars manufactured with fine recycled concrete aggregates. Comput. Chem. Eng..

[67] Braga M., de Brito J., Veiga R. (2012). Incorporation of fine concrete aggregates in mortars. Constr. Build. Mater..

[68] Vieira T., Alves A., de Brito J., Correia J., Silva R. (2016). Durability-related performance of concrete containing fine recycled aggregates from crushed bricks and sanitary ware. Mater. Des..

[69] Corinaldesi V., Moriconi G. (2009). Behaviour of cementitious mortars containing different kinds of recycled aggregate. Constr. Build. Mater..

[70] Medina C., de Rojas M.S., Frías M. (2012). Reuse of sanitary ceramic wastes as coarse aggregate in eco-efficient concretes. Cem. Concr. Compos..

[71] Martínez P.S., Cortina M.G., Martínez F.F., Sánchez A.R. (2016). Comparative study of three types of fine recycled aggregates from construction and demolition waste (CDW), and their use in masonry mortar fabrication. J. Clean. Prod..

[72] Jiménez C., Aponte D., Vázquez E., Barra M., Valls S. (2013). Equivalent Mortar Volume (EMV) Method for Proportioning Recycled Aggregate Concrete: Validation under the Spanish Context and Its Adaptation to Bolomey Methodology for Concrete Proportioning. Mater. Constr..

[73] Farinha C., de Brito J., Veiga R. (2015). Incorporation of fine sanitary ware aggregates in coating mortars. Constr. Build. Mater..

[74] Wild S., Gailius A., Hansen H., Pederson L., Szwabowski J. (1997). Pozzolanic properties of a variety of European clay bricks. Build. Res. Inf..

[75] Liu Q., Tong T., Liu S., Yang D., Yu Q. (2014). Investigation of using hybrid recycled powder from demolished concrete solids and clay bricks as a pozzolanic supplement for cement. Constr. Build. Mater..

[76] Oliveira R., de Brito J., Veiga R. (2015). Reduction of the cement content in rendering mortars with fine glass aggregates. J. Clean. Prod..

[77] Jesus S., Maia C., Farinha C.B., de Brito J., Veiga R. (2019). Rendering mortars with incorporation of very fine aggregates from construction and demolition waste. Constr. Build. Mater..

[78] Braga M., de Brito J., Veiga R. (2014). Reduction of the cement content in mortars made with fine concrete aggregates. Mater. Struct..

